# Different systolic blood pressure targets for people with history of stroke or transient ischaemic attack: PAST-BP (Prevention After Stroke—Blood Pressure) randomised controlled trial

**DOI:** 10.1136/bmj.i708

**Published:** 2016-02-25

**Authors:** Jonathan Mant, Richard J McManus, Andrea Roalfe, Kate Fletcher, Clare J Taylor, Una Martin, Satnam Virdee, Sheila Greenfield, F D Richard Hobbs

**Affiliations:** 1 Primary Care Unit, Department of Public Health and Primary Care, Strangeways Research Laboratory, University of Cambridge, Cambridge CB1 8RN, UK; 2 Nuffield Department of Primary Care Health Sciences, NIHR School for Primary Care Research, University of Oxford, Oxford, OX2 6GG, UK; 3 Primary Care Clinical Sciences, University of Birmingham, Birmingham, B15 2TT, UK; 4 School of Clinical and Experimental Medicine, University of Birmingham

## Abstract

**Objective** To assess whether using intensive blood pressure targets leads to lower blood pressure in a community population of people with prevalent cerebrovascular disease.

**Design** Open label randomised controlled trial.

**Setting** 99 general practices in England, with participants recruited in 2009-11.

**Participants** People with a history of stroke or transient ischaemic attack whose systolic blood pressure was 125 mm Hg or above.

**Interventions** Intensive systolic blood pressure target (<130 mm Hg or 10 mm Hg reduction from baseline if this was <140 mm Hg) or standard target (<140 mm Hg). Apart from the different target, patients in both arms were actively managed in the same way with regular reviews by the primary care team.

**Main outcome measure** Change in systolic blood pressure between baseline and 12 months.

**Results** 529 patients (mean age 72) were enrolled, 266 to the intensive target arm and 263 to the standard target arm, of whom 379 were included in the primary analysis (182 (68%) intensive arm; 197 (75%) standard arm). 84 patients withdrew from the study during the follow-up period (52 intensive arm; 32 standard arm). Mean systolic blood pressure dropped by 16.1 mm Hg to 127.4 mm Hg in the intensive target arm and by 12.8 mm Hg to 129.4 mm Hg in the standard arm (difference between groups 2.9 (95% confidence interval 0.2 to 5.7) mm Hg; P=0.03).

**Conclusions** Aiming for target below 130 mm Hg rather than 140 mm Hg for systolic blood pressure in people with cerebrovascular disease in primary care led to a small additional reduction in blood pressure. Active management of systolic blood pressure in this population using a <140 mm Hg target led to a clinically important reduction in blood pressure.

**Trial registration** Current Controlled Trials ISRCTN29062286.

## Introduction

Stroke accounts for about 10% of deaths internationally and for more than 4% of direct healthcare costs in developed countries.^1^ If other resources, such as lost productivity, benefit payments, and informal care costs, are taken into account, the total costs double. For example, in the United Kingdom, annual care costs are around £4.4bn (€5.7bn; $6.4bn), but total costs are £9bn a year.^2^ More than 20% of strokes are recurrent events,^3^ and if one also takes into account previous history of transient ischaemic attack (TIA), this figure rises to about 30%.^1^ Therefore, secondary prevention has a major potential role to play in reducing both morbidity and costs of stroke care. Hypertension is a key risk factor for stroke. A 20 mm Hg difference in usual systolic blood pressure is associated with a 60% lower risk of death from stroke in someone aged 50-70 years and a 50% lower risk in someone aged 70-79.^4^

The PROGRESS trial showed that treatment to lower blood pressure in people who have had a stroke or TIA reduces the risk of further stroke.^5^ However, the best way to apply this evidence in clinical practice is debated.^6^ ^7^ In particular, uncertainty exists about how intensively to lower blood pressure in people who have had a stroke or TIA.^8^ A post hoc observational analysis of the PROFESS trial found that people with recent ischaemic stroke whose systolic blood pressure was less than 130 mm Hg had a higher risk of vascular events than those with a blood pressure between 1300 and 140 mm Hg.^9^ Conversely, participants in PROGRESS whose baseline systolic blood pressure was 120-140 mm Hg and who were randomised to combination therapy had a significantly reduced risk of stroke.^10^ The SPS3 trial of different blood pressure targets in younger (mean age 63 years) patients with recent lacunar stroke found a non-significant 19% reduction in risk of stroke after one year in people treated with a systolic blood pressure target of less than 130 mm Hg compared with a 130-149 mm Hg target.^11^ Recent guidelines have drawn different conclusions from the evidence base; the European guidelines recommend a target systolic blood pressure of 140 mm Hg (or higher),^12^ and British guidelines recommend a target of 130 mm Hg.^13^

In view of these controversies, the Prevention After Stroke—Blood Pressure (PAST-BP) study compared two different targets for blood pressure lowering after stroke or TIA in people recruited from a prevalent primary care population.^14^ The aim was to determine whether setting a more intensive target in primary care would lead to a lower blood pressure, as a prelude to a trial powered to detect whether such a strategy would lead to a reduction in recurrence of stroke.

## Methods

### Participants

The methods used in PAST-BP have been reported in detail elsewhere.^14^ PAST-BP was an individually randomised trial in which participants were allocated to either an intensive blood pressure target (<130 mm Hg or a 10 mm Hg reduction if baseline pressure was <140 mm Hg) or a standard target (<140 mm Hg). Patients were recruited from 106 general practices (of which 99 contributed at least one patient) in England during 2009-11. Patients were considered for inclusion if they were on the practice’s TIA/stroke register. They were excluded if their baseline systolic blood pressure was less than 125 mm Hg, they were already taking three or more antihypertensive agents, they had a greater than 20 mm Hg postural change in systolic blood pressure on standing, they were already being treated to a 130 mm Hg systolic blood pressure target, they were unable to provide informed consent, or there was insufficient corroborative evidence that they had had a stroke or TIA. Potentially eligible participants were identified using a search of the general practice’s clinical computer system. A general practitioner reviewed this list to exclude patients for whom a study invitation would be inappropriate. The remainder were sent a letter inviting them to attend a study clinic appointment held at their general practice by a research nurse, where written informed consent was obtained.

### Randomisation and masking

The central study team at the University of Birmingham randomised patients, with minimisation based on age, sex, diabetes mellitus, atrial fibrillation, baseline systolic blood pressure, and general practice. The research nurse ascertained treatment allocation either by telephone or online.

Neither participants nor clinicians were blinded to treatment allocation. A research nurse who was not otherwise involved in the patient’s care obtained the primary outcome measure (blood pressure) by using an automated sphygmomanometer.

### Procedures

Patients randomised to the intensive arm were given a target systolic blood pressure of below 130 mm Hg or a target reduction of 10 mm Hg if their baseline blood pressure was between 125 and 140 mm Hg. The target in the standard arm was less than 140 mm Hg, irrespective of baseline blood pressure. Apart from the different blood pressure targets, the management of blood pressure was the same in both groups and was carried out by a practice nurse (to monitor blood pressure) and a general practitioner (responsible for modifying blood pressure treatment). Patients whose systolic blood pressure at baseline was above target (everyone in the intensive arm and those patients in the standard arm whose blood pressure was ≥140 mm Hg) had their antihypertensive treatment reviewed by their general practitioner. A practice nurse would see all patients at three month intervals (if their blood pressure was below target when previously measured) or after one month (if previous blood pressure was above target) and refer to the general practitioner if the blood pressure was above target. The protocol required no formal down-titration of treatment if blood pressure was below target, but general practitioners had discretion to change or reduce treatment in the light of symptoms attributable to blood pressure drugs. We provided general practitioners with treatment protocols that reflected the national guidelines for blood pressure lowering in operation at the time of the trial.^15^ In both arms of the trial, the general practitioners had access to a computer based algorithm that actively suggested drugs and dosage if the participant was above target. Follow-up ceased if the participant had a major cardiovascular event.

The primary outcome was change in systolic blood pressure between baseline and one year. Participants had blood pressure measured by a research nurse (separate from the practice nurse’s measurements described above) at baseline and at six and 12 months. Blood pressure was measured using a British Hypertension Society validated automated electronic monitor supplied and validated for the study.^16^ Blood pressure was measured in a standardised way, with the patient seated for five minutes and then six measurements taken at one minute intervals. The primary outcome was the average of the second and third measurements.

Secondary measures of blood pressure included diastolic blood pressure at six and 12 months, systolic blood pressure at six months, and proportion achieving target blood pressures at 12 months. For the systolic blood pressure, we also calculated the means of readings 2-6 and 5-6 to look for any differential effects with regard to habituation to blood pressure measurement.

We identified clinical events through review of the general practice record at 12 months. These comprised major cardiovascular events (composite of fatal and non-fatal stroke, myocardial infarction, fatal coronary heart disease, or other cardiovascular death), emergency hospital admissions, and deaths. Participants were flagged for mortality at the NHS Central Register. Side effects were assessed through the use of standard questionnaires.^14^

### Statistical analysis

We estimated that a sample size of 305 patients in each group would detect a 5 mm Hg difference in systolic blood pressure between groups with 90% power at a significance level of 5% assuming a standard deviation of 17.5 mm Hg, 10% loss to follow-up, 5% mortality, and 10% major vascular events.^5^ ^7^ We used mixed models for the primary analysis, adjusting for baseline blood pressure, age group (<80 years, ≥80 years), sex, diabetes mellitus, atrial fibrillation, and practice (as a random effect). The principal analysis was a complete case analysis. We also explored the potential effects of missing values by the use of three approaches: multiple imputation, group mean, and last available value. Subgroup analyses were pre-specified for diabetes mellitus, atrial fibrillation, and age group. In addition, we did a subgroup analysis by baseline systolic blood pressure (<140 mm Hg, ≥140 mm Hg). We compared the number of consultations, treatment changes, and side effects by using generalised mixed modelling, adjusting for the same variables as in the primary outcome. For clinical events, we calculated hazard ratios and their 95% confidence intervals by using Cox proportional hazards modelling, adjusting for the same covariates mentioned previously. We checked the proportional hazard assumption with Schoenfeld residual plots and by including interaction terms in the model (for each term by time). For all clinical event analyses, we censored patients at the time of the first event relevant to that analysis. Thus, if a patient had more than one emergency hospital admission, only the first one would be counted. We used SAS 9.2 and Stata 12 for analyses.

### Patient involvement

The study was discussed by a stroke survivor group who agreed that it was an important research question and that blood pressure was an important outcome for them. Patients were involved in developing plans for recruitment and design of the study through representation on the Trial Steering Committee. No patients were asked to advise on interpretation or writing up of the results. We plan to disseminate the results of the research to the relevant patient community through local and nationally organised stroke groups.

## Results

Figure 1 shows the trial profile; 529 patients from 99 general practices (range 1-16 per practice) entered the trial, and 84 patients withdrew from the trial in the 12 months after randomisation (52 (20%) in the intensive target arm and 32 (12%) in the standard target arm; P=0.02). Primary outcome data were available for 379 participants at one year follow-up (182 (68%) in the intensive target arm and 197 (75%) in the standard target arm). All patients were followed up for clinical events and deaths**.**Table 1 shows patients’ characteristics at baseline. About a quarter of participants were not taking any blood pressure lowering treatment at randomisation (76 in intensive arm; 63 in standard arm). For half of the participants, the index event was a TIA. Just under 20% of participants reported at least moderate disability (modified Rankin score of three or more). There were no important differences in characteristics between participants who did and did not have blood pressure recorded at 12 months (table 1).

**Figure f1:**
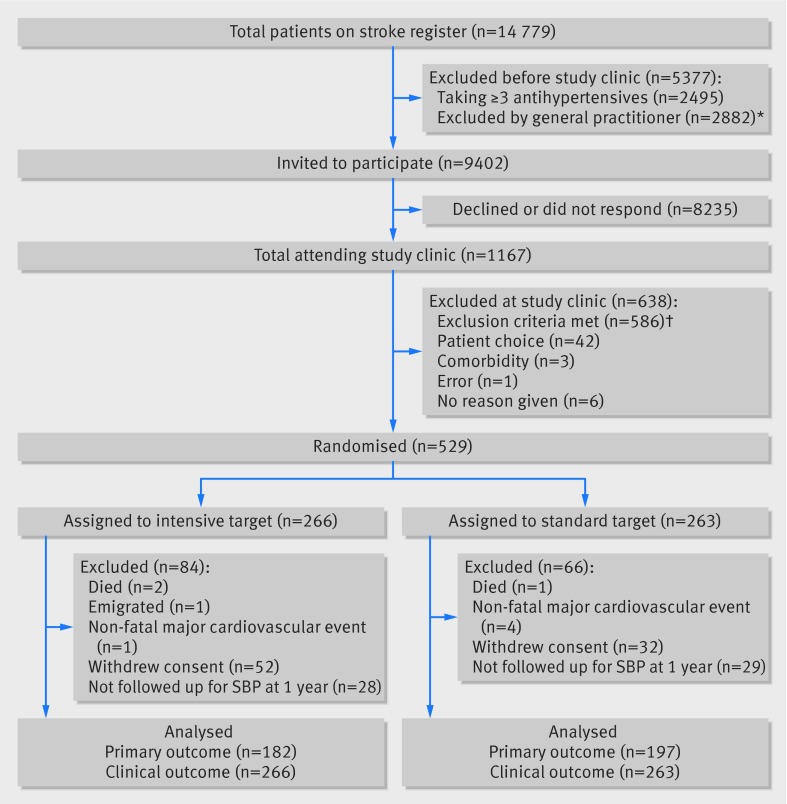
**Fig 1** Trial profile. *Reasons given: patient was housebound or in nursing home (957; 33%); would be unable to provide consent (338; 12%); comorbidity (216; 7%); blood pressure too low (199; 7%); at risk of falling (164; 6%); insufficient evidence of stroke/transient ischaemic attack (98; 3%); already being treated to 130 mm Hg target (71; 2%); other patient related factors (69; 2%); patient choice (54; 2%); terminally ill (48; 2%); deceased or left practice (41; 1%); participating in another trial (9); no reason given (618; 21%). †Blood pressure <125 mm Hg (447); lack of corroborative evidence of stroke/transient ischaemic attack (60); taking ≥3 antihypertensives (51); orthostatic hypotension (22); already being treated to lower blood pressure target (4); unable to provide informed consent (2). SBP=systolic blood pressure

**Table 1 tbl1:** Baseline characteristics. Values are numbers (percentages) unless stated otherwise

Characteristics	All participants			Participants with systolic blood pressure recorded at 12 months	
	Intensive target (n=266)	Standard target (n=263)		Intensive target (n=182)	Standard target (n=197)
Mean (SD) age, years	71.9 (9.1)	71.7 (9.4)		72.6 (8.3)	71.9 (9.5)
Male sex	157 (59)	156 (59)		104 (57)	125 (63)
White ethnicity	260 (98)	259 (98)		180 (99)	194 (98)
Current smoker	25 (9)	33 (13)		15 (8)	27 (14)
Mean (SD) SBP, mm Hg	142.9 (14.0)	142.2 (13.4)		143.5 (13.5)	142.2 (12.9)
SBP <140 mm Hg	128 (48)	129 (49)		79 (43)	98 (50)
SBP ≥140 mm Hg	138 (52)	134 (51)		103 (57)	99 (50)
Mean (SD) diastolic blood pressure, mm Hg	79.9 (10.0)	80.4 (9.8)		78.8 (9.3)	80.7 (10.1)
Diabetes mellitus	26 (10)	25 (10)		19 (10)	21 (11)
Atrial fibrillation	28 (11)	27 (10)		21 (12)	22 (11)
Coronary heart disease	41 (15)	46 (17)		28 (15)	35 (18)
Chronic kidney disease	26 (10)	30 (11)		19 (10)	23 (12)
Heart failure	2 (1)	7 (3)		1 (1)	6 (3)
Peripheral vascular disease	11 (4)	11 (4)		7 (4)	6 (3)
Stroke	130 (49)	122 (46)		85 (47)	95 (48)
Transient ischaemic attack only	135 (51)	141 (54)		97 (53)	102 (52)
Mean (SD) No of antihypertensive drugs	1.0 (0.8)	1.1 (0.8)		1.1 (0.8)	1.1 (0.8)
Mean (SD) No of other drugs	4.5 (2.5)	4.6 (2.6)		4.5 (2.5)	4.6 (2.6)
Mean (SD) total No of drugs	5.6 (2.8)	5.7 (2.7)		5.6 (2.7)	5.7 (2.7)
Modified Rankin scale*:					
0 or 1	135 (518)	125 (48)		98 (54)	84 (43)
2	65 (24)	69 (26)		42 (23)	57 (29)
3 or 4	47 (18)	51 (19)		29 (16)	42 (21)

SBP=systolic blood pressure.

*Data missing for 19 patients in intensive arm and 18 in standard arm (all participants) and for 13 patients in intensive arm and 14 in standard arm (participants with 12 month systolic blood pressure).

The intensive target arm was associated with significantly more consultations for blood pressure control with the general practitioner (median visits 2 *v* 1; P<0.001) and practice nurse (median 3 *v* 2; P=0.002) than the standard target arm. This higher consultation rate led to more intensifications of blood pressure treatment (458 *v* 278; P<0.001) and more changes due to side effects (77 *v* 30; P<0.001). However, patients were also less likely to have their blood pressure treatment increased after review by the general practitioner when the blood pressure was above target in the intensive arm (109 *v* 57; P=0.005) (table 2). The three factors that contributed most to this difference were symptoms attributed to blood pressure drugs, blood pressure only just above target, and patient not wanting treatment intensified. At the end of the study, the number of antihypertensive drugs that patients were taking had increased by a similar amount in both arms (mean number of antihypertensive drugs 2.1 in intensive arm and 1.9 in standard arm; P=0.13).

**Table 2 tbl2:** Reasons given by general practitioner for not increasing blood pressure treatment after patient referred by practice nurse with blood pressure above target

Reason	Intensive target (n=109)	Standard target (n=57)
Other blood pressure readings (eg, home readings) taken into account	17	20
Patient did not want treatment intensified	22	13
Decision taken to re-measure blood pressure at future time	19	12
Symptoms attributed to blood pressure treatment	24	5
Blood pressure only just above target	14	2
Patient had not been taking pills	9	5
Blood pressure reading attributed to anxiety of patient	3	8
Changes to drug treatment already made	4	2
Postural hypotension	3	2
Awaiting specialist advice/test results	5	–
Intercurrent illness	3	–
Patient too old for further increases in treatment	1	2
Change in lifestyle advocated rather than change in drugs	–	1

Reason was given for 164/166 non-intensification decisions. Numbers add up to more than 164, as in some cases two reasons were given.

Treatment to a more intensive target was associated with a significantly greater reduction in systolic blood pressure at 12 months (primary outcome) (table 3). Systolic blood pressure was reduced by 16 mm Hg in the intensive target arm and by 13 mm Hg in the standard target arm. This difference persisted when we calculated it by using the mean of the fifth and sixth readings (−3.2 (95% confidence interval −5.8 to −0.64) mm Hg) or the mean of the second to sixth readings (−3.3 (−5.8 to −0.67) mm Hg) (supplementary table A). When we took account of the missing values by using multiple imputation, the effect size was −3.2 (−5.7 to −0.65) mm Hg (see supplementary table B for results of other methods). The blood pressure target (that is, <130 mm Hg or a 10 mm Hg reduction for those with a baseline systolic blood pressure <140 mm Hg) at one year was achieved in 93 (51%) patients in the intensive arm. Proportions achieving a systolic blood pressure below 140 mm Hg were similar in the two arms (150/182 (82%) *v* 161/197 (82%); P=0.59), as were those achieving a systolic blood pressure below 130 mm Hg (103/182 (57%) *v* 107/197 (54%); P=0.36). We found no evidence of a significant difference in effectiveness of using an intensive blood pressure target in any subgroup of patients (fig 2).

**Table 3 tbl3:** Systolic and diastolic blood pressure in intensive target and standard target groups

	Mean (SD) blood pressure (mm Hg)				Mean (SD) difference from baseline (mm Hg)			Effect size: mm Hg (95% CI)*	
	Baseline	6 months	12 months		6 months	12 months		6 months	12 months
**Systolic blood pressure**									
Intensive target†	143.5 (13.5)	125.7 (14.5)	127.4 (14.8)		−17.3 (16.7)	−16.1 (15.0)		−4.12 (−6.84 to -1.40)	−2.94 (−5.68 to −0.21)
Standard target‡	142.2 (12.9)	129.3 (14.6)	129.4 (14.8)		−12.7 (16.7)	−12.8 (17.2)		–	–
**Diastolic blood pressure**									
Intensive target†	78.8 (9.3)	73.1 (10.3)	72.0 (9.0)		−6.5 (10.7)	−6.8 (9.1)		−1.14 (−2.86 to 0.58)	−1.63 (−3.10 to −0.15)
Standard target‡	80.7 (10.1)	74.6 (9.8)	74.4 (8.9)		−6.1 (9.7)	−6.3 (9.4)		–	–

*Adjusted for baseline blood pressure, age group (<80, ≥80 years), sex, diabetes mellitus, atrial fibrillation, and general practice (random effect).

†Blood pressure data for 193 intensive target patients at six months and 182 at 12 months.

‡Blood pressure data for 198 standard target patients at six months and 197 at 12 months.

**Figure f2:**
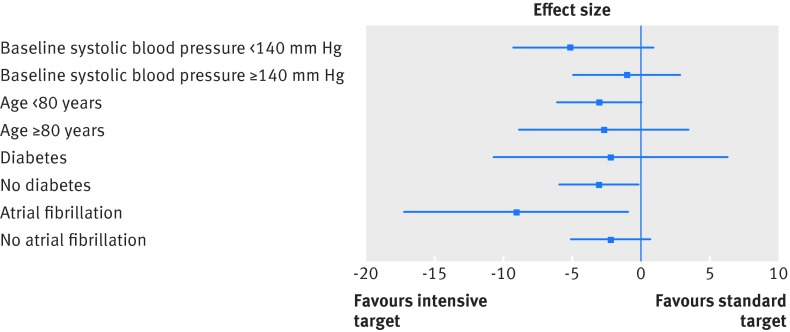
**Fig 2** Effect of intensive versus standard target on systolic blood pressure at 12 months for different patient subgroups, adjusted for baseline blood pressure, age group (<80, ≥80 years), sex, diabetes mellitus, atrial fibrillation, and general practice (random effect)

One major cardiovascular event occurred in the intensive target arm (a non-fatal myocardial infarction) and five in the standard care arm (three strokes, one non-fatal myocardial infarction, and one cardiovascular death) (hazard ratio 0.19, 95% confidence interval 0.02 to 1.87; P=0.16). Two deaths occurred in the intensive target arm and one in the standard target arm. The risk of emergency admission was 12.8% per year in the intensive target arm and 7.8% per year in the standard target arm (hazard ratio 1.56, 0.84 to 2.93; P=0.16). Two admissions in each arm were related to falls. Apart from TIA (responsible for five admissions in the standard target arm and three admissions in the intensive target arm) and stroke, no single diagnosis accounted for more than two admissions. Table 4 shows the most common symptoms at 12 months by treatment allocation. There were no significant differences between the two groups.

**Table 4 tbl4:** Most frequent symptoms at 12 months

Symptom	No (%)		Effect size: odds ratio* (95% CI)	P value
	Intensive target arm	Standard target arm		
Pain	93/163 (57)	89/173 (51)	1.17 (0.75 to 1.84)	0.48
Breathlessness	53/148 (36)	49/158 (31)	1.17 (0.72 to 1.92)	0.53
Fatigue	75/149 (50)	88/163 (54)	0.81 (0.51 to 1.28)	0.36
Stiff joints	93/162 (57)	99/176 (56)	0.94 (0.59 to 1.49)	0.80
Sore eyes	35/148 (24)	24/158 (15)	1.68 (0.93 to 3.04)	0.08
Wheeziness	32/163 (20)	28/175 (16)	1.24 (0.70 to 2.21)	0.46
Headaches	27/151 (18)	36/165 (22)	0.69 (0.38 to 1.24)	0.22
Sleep difficulties	56/150 (37)	66/163 (40)	0.81 (0.51 to 1.31)	0.39
Dizziness	45/164 (27)	39/173 (23)	1.24 (0.74 to 2.08)	0.42
Loss of strength	44/148 (30)	51/162 (31)	0.85 (0.51 to 1.40)	0.52
Loss of libido	47/160 (29)	50/171 (29)	1.06 (0.65 to 1.72)	0.83
Impotence	29/129 (22)	31/145 (21)	1.22 (0.65 to 2.30)	0.54
Pins and needles	54/163 (33)	44/176 (25)	1.48 (0.91 to 2.41)	0.11
Cough	40/144 (28)	49/160 (31)	0.86 (0.51 to 1.44)	0.57
Swelling of legs/ankles	51/162 (31)	49/177 (28)	1.10 (0.67 to 1.81)	0.70
Dry mouth	34/147 (23)	36/161 (22)	0.98 (0.57 to 1.70)	0.95

*Adjusted for baseline systolic blood pressure, age group (<80, ≥80 years), sex, diabetes mellitus, atrial fibrillation, and general practice (random effect).

## Discussion

We found that aiming for a target systolic blood pressure of below 130 mm Hg or a 10 mm Hg reduction from baseline if this was below 140 mm Hg in a primary care population with prevalent cerebrovascular disease led to a lower systolic blood pressure than if a target of below 140 mm Hg target was aimed for. However, the difference was small (about 3 mm Hg) and was associated with increased workload (one extra consultation a year each for general practitioners and nurses). The intensive target arm was not associated with more side effects as measured at follow-up, but more changes to treatment occurred because of side effects during the trial. More people from the intensive target arm withdrew consent for the trial, and this might have reflected unwillingness to persevere with the increased treatment regimen. Perhaps the most important finding was the greater than 10 mm Hg reductions in mean systolic blood pressure in both arms of the study, so that more than 80% of participants in each arm had achieved a blood pressure of below 140 mm Hg by the end of the trial, compared with less than 50% at baseline.

### Strengths and weaknesses of study

Blood pressure at 12 months was not available for 28% of patients randomised. This reflected a high number of withdrawals from the study, with some differential loss to follow-up in the intensive target arm. However, when we imputed missing values by using multiple imputation (the most robust method), the difference in achieved blood pressure between arms at one year was very similar to that observed. Although we did not achieve our sample size, in the event our trial was adequately powered, as the observed standard deviation in blood pressure was less than we had anticipated in our sample size calculation. This is reflected in the statistical significance of the small difference in observed blood pressure between arms. Nevertheless, the upper limit of the confidence interval around the difference between arms at one year was 5.68 mm Hg, which would be regarded as a clinically important effect. Only 4% of patients on general practice stroke/TIA registers participated in the trial. Participants had a low prevalence of disability for a prevalent cerebrovascular disease population, were younger than typical patients in primary care with a history of cerebrovascular disease, and over-represented people with a history of TIA only.^7^ It is likely, therefore, that the more intensive target would have been even harder to achieve if the trial population was more representative of people with prevalent cerebrovascular disease. The trial represents a post-stroke primary care population managed by generalists rather than a selective hospital/outpatient population managed by specialists. The outcome measure was not blinded, but a nurse not directly involved in the participant’s care obtained it by using an automated sphygmomanometer, so systematic recording bias is unlikely.

The standard target arm in PAST-BP was actively managed, with the support of a computer based algorithm that suggested drug changes, rather than simply receiving “usual care.” If we had used a more passive management strategy in the comparison group, we may have achieved a greater separation in systolic blood pressure between arms. In another blood pressure lowering study of patients with increased cardiovascular risk carried out by our group in the same timeframe, the standard care control arm dropped by 6 mm Hg from a similar baseline compared with 13 mm Hg in the study reported here.^17^ We used an active control because we wanted to ascertain the effect of setting different blood pressure targets and to avoid confounding that would be introduced by having different management strategies in the two arms. The target in the intensive arm was more complicated than that in the standard care arm, but we minimised the effect of this on adherence to the protocol by ensuring that the primary care staff managed all trial participants in the same way, with prompts to review treatment if blood pressure was above the individualised target.

### Comparison with other studies and interpretation

The change in mean blood pressure that we observed in the intensive target arm was very similar to that observed in the below 130 mm Hg target arm of the SPS3 trial, with both PAST-BP and SPS3 achieving a mean systolic blood pressure in the intensive arm of 127 mm Hg after one year.^11^ However, the comparison arms had different achieved blood pressures (129 mm Hg in PAST-BP versus 138 mm Hg in SPS3). This reflects the more conservative target in the higher target arm of SPS3 (130-149 mm Hg as opposed to <140 mm Hg) and that antihypertensive treatment was reduced if blood pressure fell below target.

Most of the observed reduction in blood pressure is likely to have been mediated by increased use of antihypertensive drugs, which on average went up from one to two drugs per person over the year of the study in both arms of the trial. Alternative explanations are that habituation to blood pressure measurement occurred, leading to reduced white coat effect, or that there was regression dilution bias. However, in a blood pressure monitoring trial in a similar post-stroke population with similar mean baseline systolic blood pressure, no fall in blood pressure was observed in the control group over a 12 month period,^18^ and in the SPS3 trial (also with similar mean baseline systolic blood pressure to PAST-BP) a fall of just 4 mm Hg was seen in the 140 mm Hg target arm over the study period.^11^ This suggests that the fall of 13 mm Hg observed in the standard target arm of PAST-BP is unlikely to be primarily due to effects of regression dilution or habituation to measurement. Given that we had a relatively low systolic blood pressure inclusion criterion of 125 mm Hg or above, important regression dilution bias would not be anticipated in this study.

Only 51% of patients in the intensive target arm of PAST-BP achieved their target blood pressure. Both patients’ wishes and general practitioners’ decision making led to treatment not being intensified when blood pressure was above target (table 2). Greater reluctance to lower blood pressure when near target, higher attribution of symptoms to blood pressure treatment (table 2) despite an absence of objective evidence of increased symptoms (table 4) in the intensive target arm, and greater reluctance of patients to increase treatment hint at the difficulties faced in achieving lower blood pressure targets in clinical practice.^19^ This impression of practical difficulty is reinforced by the significantly higher proportion of participants that withdrew from the trial in the intensive arm. Although reported side effects and symptoms were similar in the two arms, and serious adverse events were infrequent (two admissions for falls in each arm), significantly more changes to treatment needed to be made because of side effects in the intensive target arm.

### Implications

Recent evidence from SPRINT and a systematic review highlight the benefits of intensive blood pressure lowering.^20^ ^21^ In some blood pressure target trials such as SPRINT and SPS3, the trial design maximised the achieved difference in blood pressure between the two arms, with the less intensive arm having a target range rather than simply a below 140 mm Hg systolic target, and with treatment being reduced if blood pressure fell below the target range. This is an appropriate design for an explanatory trial designed to test the question does lowering blood pressure reduce risk of cardiovascular events? In our pragmatic trial, which sought to test the effect of different blood pressure targets as they would be used in clinical practice, the protocol did not stipulate a reduction in blood pressure treatment if the blood pressure was below target and the control arm was actively managed to achieve a target blood pressure below 140 mm Hg. As a result of this, and of reluctance on the part of both clinicians and patients to instigate all increases in blood pressure treatment in the intensive group, the achieved difference in blood pressure between the two arms was small. Nevertheless, we found that active management was associated with clinically important reductions in blood pressure in both arms—the 13 mm Hg reduction achieved in the below 140 mm Hg arm equates to more than 40% and 20% reduction in the risk of stroke and coronary heart disease respectively.^22^ The reduction in blood pressure in our less intensive arm was similar to that achieved in the active arms of other blood pressure lowering trials and more than in their control groups.^11^ ^17^ The additional resources needed to achieve the additional 3 mm Hg lower blood pressure in the intensive target arm might be better spent in increasing the proportion of people with stroke in primary care who have a systolic blood pressure below 140 mm Hg. Given this conclusion, we did not feel that a pragmatic trial powered to detect a difference in cardiovascular endpoints achieved using an intensive target in primary care was warranted. Furthermore, the ongoing ESH-CHL SHOT trial will provide important data on whether intensive blood pressure lowering reduces cardiovascular events in people with stroke (who were excluded from the SPRINT trial).^23^ The explanatory trial design is likely to lead to clear differences in achieved blood pressure in the treatment arms and confirm whether intensive blood pressure lowering reduces cardiovascular endpoints in the post-stroke population.

**What is already know on this topic**

Decreasing blood pressure after stroke is associated with a lower risk of stroke recurrence, but uncertainty exists about what the target blood pressure should be
One trial in people with recent lacunar stroke found that a systolic blood pressure target of <130 mm Hg was associated with a non-significant reduction in stroke compared with a target of 130-149 mm Hg
No trials of different blood pressure targets after stroke have been carried out in primary care settings

**What this study adds**

Patients set a target of <130 mm Hg or a 10 mm Hg reduction if initial blood pressure was <140 mm Hg achieved lower systolic blood pressures than those set a target of <140 mm Hg
However, the difference was small (3 mm Hg) in the context of the reduction in blood pressure observed in both arms (13 mm Hg and 16 mm Hg)
Active management of blood pressure after stroke/transient ischaemic attack is more important than the target that is set

## Web Extra

Extra material supplied by the author

## Notes

Ethical approval: Ethical approval was provided by the Warwickshire Research Ethics Committee (reference 08/H1211/121). All participants gave written informed consent.

Transparency: The lead author (the manuscript’s guarantor) affirms that this manuscript is an honest, accurate, and transparent account of the study being reported; that no important aspects of the study have been omitted; and that any discrepancies from the study as planned (and, if relevant, registered) have been explained.

Data sharing: No additional data available.
