# Supplementary material for: Different systolic blood pressure targets for people with history of stroke or transient ischaemic attack: PAST-BP (Prevention After Stroke—Blood Pressure) randomised controlled trial
Source: BMJ. 2016 Feb 25;352:i708. doi: 10.1136/bmj.i708 (PMC4770816; doi:10.1136/bmj.i708)
Supplement: Supplementary file 1 — Supplementary tables [file manj029605.ww1_default.pdf]

**Supplementary tables:**

| | | Mean blood pressure (mm Hg) | | Mean difference from baseline (mm Hg) | Effect size (mm Hg, 95% CI)† |
|---|---|---|---|---|---|
| | | Baseline | 12 months | | |
| | | **Systolic blood pressure** | | | |
| **Average of 5th and 6th readings** | Intensive target | 137.3 (13.5) | 122.7 (14.9) | -14.7 (16.0) | -3.24 (-5.8 to -0.64) |
| | Standard target | 135.5 (13.3) | 124.9 (13.2) | -11.0 (16.6) | .. |
| **Average of 2nd to 6th readings** | Intensive target | 140.0 (13.0) | 125.0 (14.7) | -15.1 (14.9) | -3.25 (-5.8 to -0.67) |
| | Standard target | 138.4 (12.7) | 127.2 (13.6) | -11.6 (16.2) | .. |

†Adjusted for baseline blood pressure, age group (<80, ≥80), gender, diabetes mellitus, atrial fibrillation and general practice (random effect)

**Table A: Systolic blood pressure in intensive target and standard target groups**

| | | Mean blood pressure (mm Hg) | | Mean difference from baseline (mm Hg) | Effect size (mm Hg, 95% CI)† |
|---|---|---|---|---|---|
| | | Baseline | 12 months | | |
| **Imputation method** | | **Systolic blood pressure** | | | |
| Multiple imputation | Intensive target | 142.9 (13.9) | 126.8 (14.9) | -16.1 (15.4) | -3.19 (-5.72 to -0.65) |
| | Standard target | 142.2 (13.3) | 129.6 (14.7) | -12.6 (17.4) | .. |
| Group mean | Intensive target | 142.9 (14.0) | 127.4 (12.2) | -15.5 (15.0) | -2.33 (-4.33 to -0.32) |
| | Standard target | 142.2 (13.4) | 129.4 (12.8) | -12.8 (16.6) | .. |
| Last value carried forward | Intensive target | 142.9 (14.0) | 130.0 (15.4) | -12.9 (15.5) | -1.79 (-4.15 to 0.57) |
| | Standard target | 142.2 (13.4) | 131.3 (15.6) | -10.8 (16.8) | |

†Adjusted for baseline blood pressure, age group (<80, ≥80), gender, diabetes mellitus, atrial fibrillation and general practice (random effect)

**Table B: Systolic blood pressure in intensive target and standard target groups with imputation of missing data**
